# Controlling the Cooling Rate of Hydrothermal Synthesis to Enhance the Supercapacitive Properties of β-Nickel Hydroxide Electrode Materials

**DOI:** 10.3390/ma16165576

**Published:** 2023-08-11

**Authors:** Yang-Ming Lu, Sheng-Huai Hong

**Affiliations:** Department of Electrical Engineering, National University of Tainan, Tainan 7005, Taiwan

**Keywords:** β-nickel hydroxide, supercapacitors, electrochemical materials, electrode materials, capacitance characteristics

## Abstract

The demand for power storage devices with good quality, fast charging and high energy density is becoming more and more urgent in today’s electronic technology. For batteries and traditional capacitors, it is an insurmountable challenge to combine fast charging and discharging, large capacitance and long-life properties. The characteristics of supercapacitors can meet all the above requirements at the same time. In this study, a simple one-step hydrothermal method was successfully used to grow β-nickel hydroxide nanocone particles directly on the 3D foamed nickel substrate as a working electrode material for supercapacitors. After growing β-nickel hydroxide crystals on 3D foamed nickel substrate, by controlling the cooling rate, a well-crystalized β-nickel hydroxide with good capacitance characteristics can be obtained. Cyclic voltammetry (CV), galvanostatic charge–discharge (GCD) and electrochemical impedance spectroscopy (EIS) were used to analyze the capacitance characteristics of the β-nickel hydroxide electrode. The research results show that the specific capacitance value of the β-Ni(OH)_2_/3D nickel foam electrode material prepared at the cooling rate of 10 °C/h can reach 539 F/g with the charge–discharge test at a current density of 3 A/g. After 1000 continuous charge and discharge cycles, the material still retains 94.1% of the specific capacitance value.

## 1. Introduction

Energy issues are always a topic of concern in the development of modern society. Non-renewable fossil fuels will run out one day, and their pollution of the environment and waste of resources cannot be ignored. Renewable energy is receiving more and more attention because it is expected to take into account both environmental protection and sustainable development. Part of the renewable energy is used directly, and the excess energy is stored in the form of electric energy. Therefore, energy storage devices with high energy-storage efficiency and environmental friendliness have attracted great attention all over the world. However, renewable energy is heavily influenced by the environment, and there are still great challenges in power generation and electric energy storage technologies. Therefore, it is particularly important to develop energy storage devices with fast charging and discharging capabilities [1].

Supercapacitors have the characteristics of high power density, long cycle life, fast charge and discharge rate, wide operating temperature and low maintenance cost [2,3,4]. Their energy density is higher than that of traditional capacitors, and their power density is higher than that of lithium-ion batteries. Supercapacitors are a new type of energy storage device between lithium-ion batteries and traditional capacitors [5,6] and have great potential to replace them. Because of their excellent power density and charge–discharge rate, supercapacitors are widely used in various fields such as biomedicine [7], automobile [8], aerospace [9], military [10] and so on. In supercapacitor devices, the electrochemical performance of electrodes plays an important role in energy storage. Excellent electrode materials need to be safe and have high conductivity, high specific surface area and good stability. Therefore, knowing how to design electrode materials that are environmentally friendly, easy to prepare and have excellent performance is very important for the power storage of supercapacitors.

Although transition metal oxides or hydroxides have high capacitance, they are limited by the kinetic properties of redox reactions, and their stability is not good when charging and discharging at high rates [11,12,13,14]. To solve this problem, the active capacitor material is directly grown on the three-dimensional structure of the charge collector, which can effectively shorten the transmission and diffusion paths of electrons and ions, respectively, and improve the reactivity and utilization between the electrolyte and the electrode material [15,16,17]. Porous three-dimensional network metals are a very promising substrate, and their high surface area makes them attractive in the research of various energy storage devices such as fuel cells [18,19], lithium-ion batteries [20] and supercapacitors [21,22,23]. Three-dimensional substrates include foamed titanium, foamed copper, foamed iron, foamed cobalt and foamed nickel. Because of the excellent physical and chemical properties of foamed nickel mesh, it is the most widely used 3D mesh metal substrate [24].

It can be understood from the literature that ruthenium oxide has a very high theoretical specific capacitance (1400–2000 F/g) [25,26,27], but ruthenium is a noble metal with a high cost and scarce output, which is not conducive to commercialization. Therefore, the use of transition metal oxides as electrode materials is one of the current research directions for supercapacitors. Table 1 presents a comprehensive comparison of different transition metal oxides as supercapacitor electrode materials. Among them, the nickel-based electrode material [28] stands out among many metal materials and has attracted attention because of its relatively low price and excellent electrochemical performance. The theoretical specific capacitance of nickel hydroxide Ni(OH)_2_ is as high as 2082 F/g [29], and it is easy to prepare with a simple method. Therefore, in consideration of cost, quantity and electrochemical performance, nickel hydroxide is quite suitable as an electrode material for supercapacitors. Nickel hydroxide exists in two crystal forms, α and β. There is ion intercalation in α-Ni(OH)_2_, and the water molecules in it increase the carrier mobility during the discharge process and quickly replenish the missing carriers on the reaction surface. Therefore, α-Ni(OH)_2_ has a higher specific capacitance value than β-Ni(OH)_2_. However, α-Ni(OH)_2_ undergoes an aging phenomenon during the charging and discharging process, so its cycle life and stability are worse than those of β-Ni(OH)_2_. However, there is no ion intercalation in the β-Ni(OH)_2_ structure, resulting in a slower carrier transport rate and a smaller specific capacitance than α-Ni(OH)_2_, but its cycle life and stability are better [30]. Jingyao Qi et al. [31] obtained nickel hydroxide crystalline materials with two different preparation methods. The crystallinity characteristics were judged from the characteristic diffraction peak width and shape of the XRD. According to the analysis results, it can be reported that nickel hydroxide with poor crystallinity has poor redox reversibility, capacitance value and cycle life. From these research results, it can be seen that the crystallinity of nickel hydroxide has a considerable influence on the electrochemical performance of the electrode.

## 2. Experimental Section

A 3D nickel foam was used as the substrate to grow nickel hydroxide on it using a hydrothermal method. It was first cut into a 1 cm × 1 cm test piece. All test pieces were sequentially cleaned in acetone, isopropanol, hydrochloric acid and deionized water with an ultrasonic oscillator for 15 min to completely clean their surfaces. A simple one-step hydrothermal method was used to directly grow nickel hydroxide crystals on the surface of the 3D nickel foam test piece (substrate). The hydrothermal reaction solution was obtained by adding 2.25 mmol of nickel nitrate ((Ni(NO_3_)_2_·6H_2_O) and 4.50 mmol of urea (urea) into a beaker filled with 45 mL of deionized water.

**Figure 1:**
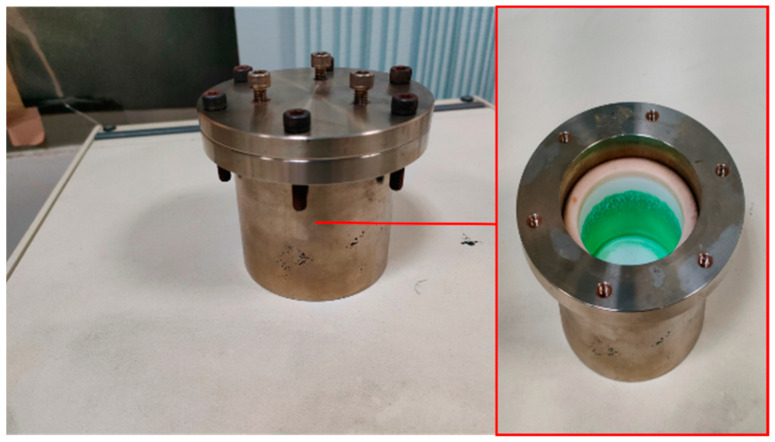
Exterior and interior of high-pressure hydrothermal kettle.

**Figure 2:**
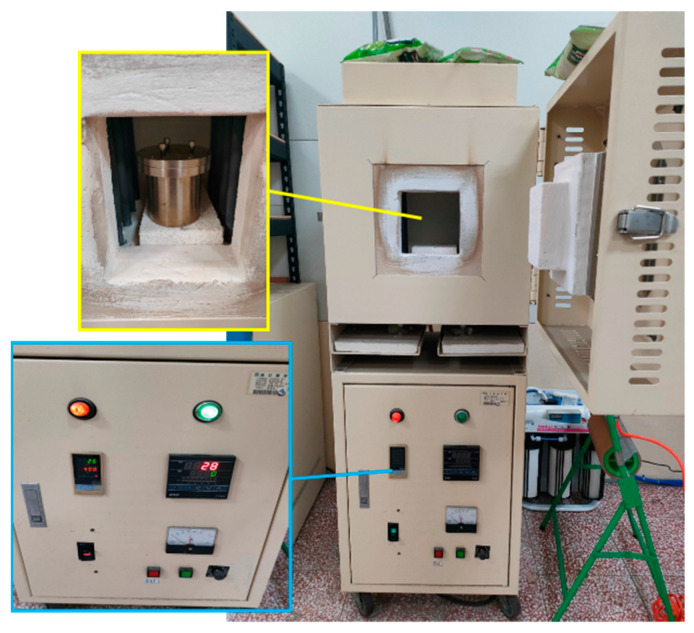
Exterior of high-temperature furnace and program controller.

All the reaction liquids were mixed with ultrasonic vibration for 40 min to obtain a light-green and clear mixed solution. This mixed solution and the pre-treated 3D nickel foam substrate were placed into a Teflon lining of a 75 mL high-pressure hydrothermal kettle (Figure 1). The kettle was heated (Figure 2) to 200 °C and kept at this high temperature for hydrothermal reaction for 6 h. The program controller was set to regulate the cooling rate of the high-temperature furnace. There were three different cooling rates of 100 °C/h, 25 °C/h and 10 °C/h used for the test pieces to cool down to room temperature. These three test pieces were named N_100_, N_25_ and N_10_, respectively.

Sun et al. used urea as an OH^-^ source to grow nickel hydroxide crystals using a hydrothermal method. In that paper, they proposed a possible mechanism for the growth of nickel hydroxide crystals [39]. The high-temperature hydrothermal growth of the nickel hydroxide crystals used in our study also has a similar growth mechanism. When the solution in the hydrothermal reaction is heated to a high temperature, a combination reaction of the Ni^2+^ and OH^−^ in the aqueous solution takes place on the surface of the 3D nickel foam and starts to nuclate Ni(OH)_2_ nanocrystals because those nuclei have high surface energy and are thermodynamically unstable. The dipole interaction between the nano-nuclei enables them to gather and form nanocrystals. Finally, those Ni(OH)_2_ nanocrystals continue to aggregate and begin to crystallize. The crystal starts to grow along the c-axis direction and gradually grows into a nanocone crystalline appearance. The crystalline appearance of the crystal is determined by the state of the solution, such as pH value, solution concentration, temperature, etc.

In this experiment, a D8 Advance ECO X-ray diffractometer was used to analyze the crystal structure of nickel hydroxide, and a HITACHI SU8000 (bought from Hitachi, Japan) high-resolution scanning electron microscope (HRSEM) was used to observe the surface morphology of the test piece. A three-electrode electrochemical system was used to measure the electrochemical properties of electrode materials (nickel hydroxide on 3D nickel foam substrates) prepared using a hydrothermal method. The three-electrode electrochemical system is composed of the following components: a 1 M KOH solution as electrolyte, 1 cm × 1 cm platinum sheet as counter electrode, Ag/AgCl electrode (keep in saturated KCl solution when not in use) as reference electrode and the well-prepared Ni(OH)_2_/3D nickel foam as working electrode.

The Admiral Squidstat Solo electrochemical instrument was used to perform cyclic voltammetry (CV), galvanostatic charge–discharge (GCD) and electrochemical impedance spectroscopy (EIS) to analyze the electrochemical properties of electrode material.

The specific capacitance value of the working electrode can be calculated by bringing the integral result of the CV measurement curve into the following formula [40]:

(1) $$C_{s}=\frac{\int \mathrm{idV}}{2\;m\left(\Delta V\right)\left(V_{s}\right)}$$

where C*_s_* is the specific capacitance value (F/g), ∫idV is the integral value of the curve, m is the mass of the electrode material (g), ΔV is the charge and discharge potential window (V) and V*_s_* is the scan rate (V/s).

The specific capacitance value of the working electrode can also be calculated by bringing the GCD analysis results into the following formula [41]:

(2) $$C_{s}=\frac{\left(\Delta t\right)\left(I\right)}{m\left(\Delta V\right)}$$

where △t is the total discharge time (*s*), I (A) is the charge and discharge current, m is the mass of the electrode material (g) and ΔV is the charge and discharge potential window (V).

## 3. Results and Discussion

After completing the hydrothermal reaction for the test pieces, the temperature was cooled to room temperature at three different cooling rates. The effect of the cooling rate on the crystallization characteristics of nickel hydroxide was investigated. Figure 1 displays the XRD patterns of N_100_, N_25_ and N_10_. Among them, the two more obvious characteristic peaks at 44.50° and 51.84° are the signals of the foamed nickel substrate.

**Figure 3:**
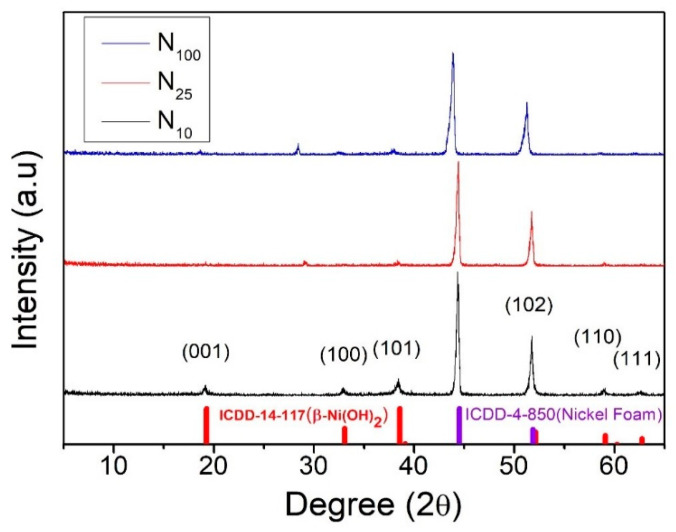
XRD patterns of nickel hydroxide cooled to room temperature at different rates.

The diffraction peak signals at 19.25°, 33.06°, 38.54°, 52.10°, 59.05° and 62.72° respectively correspond to (001), (100), (101) of β-phase nickel hydroxide (102), (110) and (111) crystal faces (JCPDS 14-0117), as shown in Figure 3.

**Figure 4:**
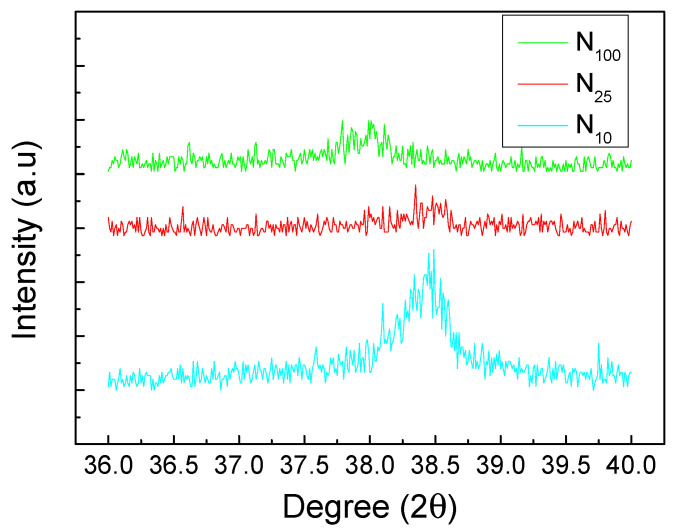
Enlarged XRD pattern of nickel hydroxide (101) diffraction peak.

This XRD analysis result (Figure 3) confirms that the nickel hydroxide prepared using the hydrothermal method in this experiment has a β-Ni(OH)_2_ crystal form. In addition, the signal intensity of the diffraction peak of β-Ni(OH)_2_ at 52.10° is the highest one, but the diffraction peak of 3D foamed nickel substrate is quite close to this peak, which is easily confused and makes it difficult to distinguish between them. The intensity of the signal peak located at 38.54° (101) is the second-strongest diffraction peak and occurred without the interference of the substrate material diffraction peak signal. Therefore, this experiment uses this diffraction peak as the main basis for subsequent analysis of the crystallization characteristics of β-Ni(OH)_2_. The (101) peak intensities of the test pieces cooled down to room temperature using different cooling rates after the hydrothermal synthesis method are significantly different. The (101) peak intensities of N_100_, N_25_ and N_10_ are 33, 73 and 253, respectively. The (101) diffraction peak of N_10_ is particularly obvious as shown in Figure 4. It can be deduced that as the cooling rate slows down, more time is provided for atoms to diffuse, which results in a more ordered crystal structure. The better the crystallization of the working electrode material, the more complete the redox reaction can be during the charge and discharge process of the supercapacitor. As a result, the specific capacitance value can be increased.

**Figure 5:**
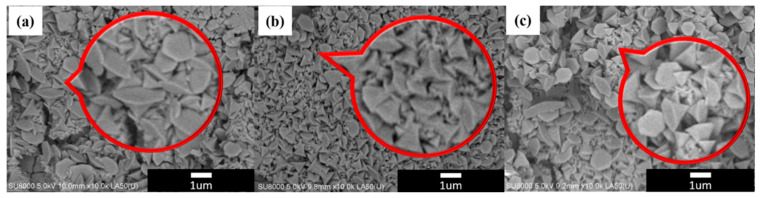
The SEM images of nickel hydroxide with different cooling rates (**a**) N_100_, (**b**) N_25_ and (**c**) N_10_. Red area is zoom-in figure of the area where it’s pointed to.

**Figure 6:**
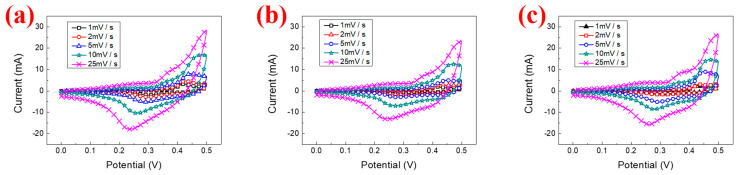
The CV curves of nickel hydroxide prepared at different cooling rates (**a**) N_100_, (**b**) N_25_ and (**c**) N_10_.

The surface microstructures of β-nickel hydroxide are shown in Figure 5. From Figure 5a–c, it can be observed that the β-nickel hydroxide films prepared at different cooling rates are all composed of cone-shaped particles ranging in size from 500 nm to 1 µm, and there is not much change in morphology. Figure 6a–c displays the CV curves of N_100_, N_25_ and N_10_ at scan rates from 1 mV/s to 25 mV/s. It can be observed that with the increase in the scan rate, the peak position of the oxidation peak gradually shifts to a high potential, while the peak position of the reduction peak gradually shifts to a low potential. This phenomenon is caused by the fact that the speed at which ions in the electrolyte migrate to the electrode surface and react with the material cannot keep up with the migration speed of electrons.

**Figure 7:**
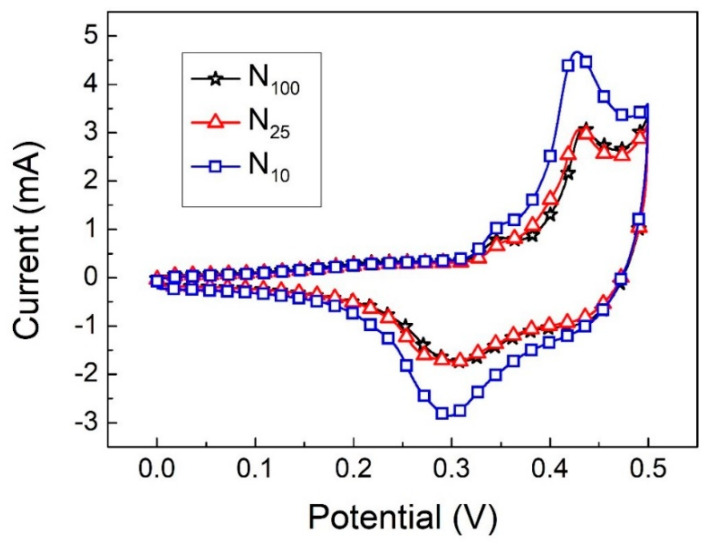
The CV curves of N_100_, N_25_ and N_10_ at a scan rate of 2 mV/s.

From the CV curve in Figure 7 (at a scan rate of 2 mV/s), the specific capacitance value was calculated according to Equation (2). The specific capacitance values of N_100_, N_25_ and N_10_ are 257.6, 281.4 and 378.9 F/g, respectively. The specific capacitance values increase as the cooling rate becomes slower, which is consistent with the inference results of XRD analysis. Because the crystallization of N10 is more obvious and orderly, it can carry out a more complete redox reaction during the charging and discharging process so that the peak current value increases, resulting in an increase in the specific capacitance value. In addition to the improvement in the specific capacitance value, the redox stability of the material can also be inferred from the ratio I_pb_/I_pa_ of the oxidation peak current (I_pb_) to the reduction peak current (I_pb_). The I_pb_/I_pa_ values of N_100_, N_25_ and N_10_ are 0.56, 0.57 and 0.61, respectively. Thus, it can be seen that the β-Ni(OH)_2_ crystallization obtained at a slower cooling rate (N10) has more complete crystallinity and performs better in terms of electrochemical redox stability.

**Figure 8:**
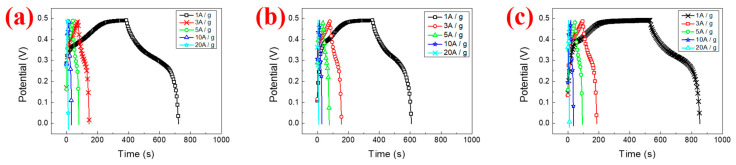
The GCD curves (**a**) N_100_, (**b**) N_25_ and (**c**) N_10_.

Figure 8a–c presents the GCD curves of N_100_, N_25_ and N_10_ at different charge–discharge current densities. It can be observed that during the charging process, a plateau interval appears in the GCD curve between 0.4 V and 0.45 V. At this time, nickel hydroxide undergoes an oxidation reaction and absorbs electrons to transform into NiOOH. During the discharge process, there is also an interval with a gentler slope between 0.3 V and 0.2 V. At this time, NiOOH undergoes a reduction reaction, transforms into Ni(OH)_2_ and emits electrons, which is the standard pseudocapacitance performance.

**Figure 9:**
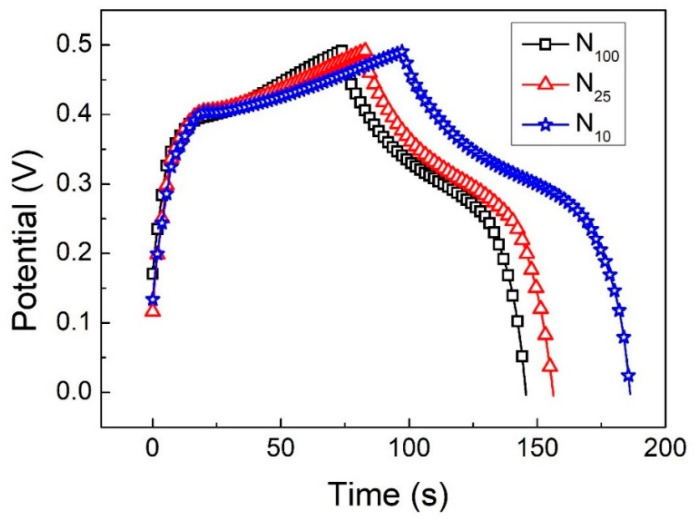
The GCD curves of N_100_, N_25_ and N_10_ at a current density of 3 A/g.

Figure 9 presents the GCD curves of the three samples under the potential window of 0–0.49 V, with a current density of 3 A/g for the charge and discharge cycles. It can be observed that under the same potential window, the charging and discharging time of N10 is longer than that of the other two. The specific capacitance values are calculated using Equation (2). N_100_, N_25_ and N_10_ have specific capacitance values of 434.6, 446.3 and 538.7 F/g, respectively. Because CV is measured by changing the potential through the potential difference, and GCD is charged and discharged by passing a current, the values measured with the two measurement methods are different. However, the results measured using CV (Figure 7) and GCD (Figure 9) tend to be consistent; that is, the specific capacitance value increases as the cooling rate becomes slower after the hydrothermal synthesis method is completed. Although the measurement methods are different, both show the same trend results. That is to say, the specific capacitance value of the supercapacitor increases as the cooling rate of the electrode material prepared using the hydrothermal synthesis method slows down.

The energy efficiency can be calculated with the following formula [42]:

$$\mathrm{Energy}\;\mathrm{efficiency}=\frac{energy\;density\left(discharge\right)}{energy\;density\left(charge\right)}\times 100$$

The calculated results are summarized in Table 2 as shown below.

From the calculated data in Table 2, it is obvious that the N_10_ test piece has the highest energy density. This result is quite consistent with the previous analysis results and inferences.

**Figure 10:**
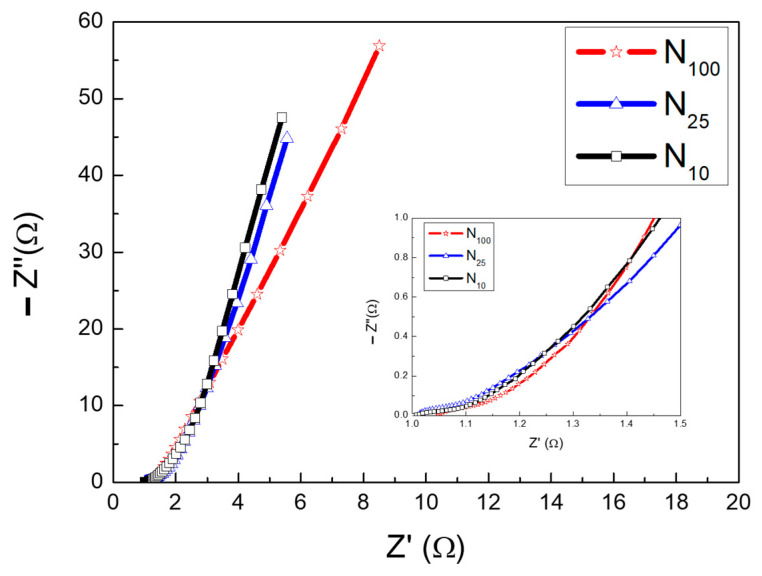
EIS Nyquist plots of N_100_, N_25_ and N_10_.

Figure 10 is the Nyquist diagram of N_100_, N_25_ and N_10_ tested using EIS. The AC impedance corresponding to different AC frequencies is divided into a real part (Z′) and an imaginary part (−Z″). The value where the curve intersects the x-axis in the high-frequency region is the equivalent series resistance (ESR), which comes from the solution impedance and the internal resistance of the electrode. In addition, a typical Nyquist diagram has a semicircle in the high-frequency region. The diameter of this semicircle is the charge transfer resistance (R_ct_). This resistance value can be used as a reference for the charge transfer speed inside the electrode. The smaller the R_ct_ value, the faster the charge transfer rate inside the electrode [43]. It is known from the literature [44] that the EIS Nyquist plot of supercapacitors is between that of typical battery materials and ideal capacitor materials. Observing an obvious semicircle in the plot is not easy. Therefore, in this experiment, we used simulation software and selected multiple points on the bending line in the high-frequency region to estimate ESR and Rct values. The ESR values of N_100_, N_25_ and N_10_ measured from the intercept of the x-axis in the plot are 1.052 Ω, 1.012 Ω and 1.010 Ω, respectively. The R_ct_ of these three specimens is calculated by fitting the software simulation to be 0.35 Ω, 0.33 Ω and 0.22 Ω, respectively. Both sets of data indicate that the internal resistance of the electrode of N10 is slightly smaller and that the internal charge transfer rate is faster. This phenomenon is consistent with the results of XRD, CV and GCD. In the low-frequency region, the slope of the straight line of N10 is larger than that of the other two, indicating that the diffusion resistance of OH^−^ radical ions in the electrolyte is lower in N_10_. This means that N10 has excellent capacitance material properties, making it easier for OH^−^ ions in the electrolyte to diffuse into the electrodes.

**Figure 11:**
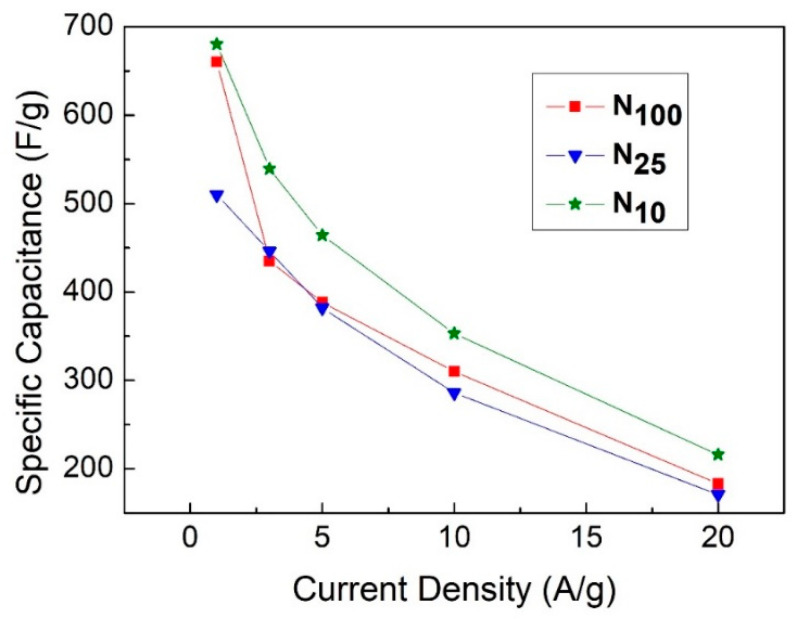
Specific capacitance values of N_100_, N_25_ and N_10_ as a function of current density.

Figure 11 is a graph showing how specific capacitance values of all the samples changed with the charge and discharge current density. It can be found that the specific capacitance of N_10_ with the slowest cooling rate is larger than that of N_100_ and N_25_ at every different current density. At a current density of 3 A/g, the specific capacitance of N_10_ is 539 F/g, and it maintains a specific capacitance of 216 F/g at a high current density of 20 A/g. Compared with N_100_ and N_25_, N_10_ has a better ability to withstand a high current when charged and discharged. This result is consistent with the XRD deduction results. Because of the more complete crystallization of N10, the carrier transport rate is faster, so it maintains excellent capacitance material characteristics under high current density.

Table 3 provides a comparison of different preparation methods of nickel hydroxide as a supercapacitor electrode material. It can be seen that using a simple one-step hydrothermal method to directly grow nickel hydroxide on the 3D nickel foam substrate is much simpler. The specific capacitance value is not inferior to that of electrode materials prepared using other processes.

**Figure 12:**
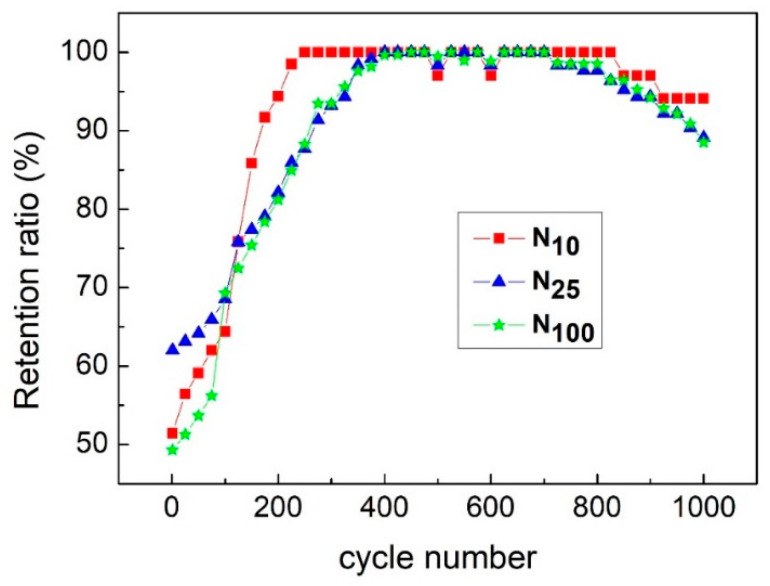
Variation in the specific capacitance values of N_100_, N_25_ and N_10_ after 1000 continuous charge and discharge cycles.

Figure 12 is a graph showing changes in the specific capacitance values of N_100_, N_25_ and N_10_ after 1000 continuous charge and discharge cycles at a charge and discharge current density of 20 A/g. It can be observed from Figure 10 that after 200 to 400 cycles, the specific capacitance value of the capacitor increases from about 50% of the original maximum specific capacitance value to 100%. This phenomenon can be attributed to the increased degree of the wetting effect between the electrode material and the electrolyte. After many charge–discharge cycles, the electrolyte more easily wets the inner surface of the porous electrode, making it easier for the ions in the electrolyte to diffuse into the electrode material. Therefore, the reaction area that can carry out redox increases, so the specific capacitance value quickly rises to its maximum specific capacitance value. After 1000 charge–discharge cycles, the specific capacitance values of N_100_, N_25_ and N_10_ can still maintain 88.5%, 89.1% and 94.1%, respectively. N10 can maintain 94.1% of the specific capacitance value, which is attributed to the slower cooling rate that makes the crystallization of β-nickel hydroxide more complete and better so that N_10_ still has excellent capacitance characteristics after a large number of charge–discharge cycles. Such an excellent capacitor charge–discharge cycle life can prove that β-nickel hydroxide has high reliability as an electrode material for supercapacitors.

## 4. Conclusions

In this experiment, β-nickel hydroxide was successfully prepared as an electrode material for supercapacitors using a simple one-step hydrothermal method. Slowing down the cooling rate of the hydrothermal process can make the crystallization of β-nickel hydroxide more complete. The hydrothermally grown β-nickel hydroxide crystalline state presents the appearance of cone-shaped particles, which effectively increases the surface area of the electrode. According to the research results, the capacitance characteristic of the test piece (N_10_) with the slowest cooling rate is the best. Under the current density of 3 A/g, the constant current charge and discharge experiment was carried out, and the specific capacitance value was 538.7 F/g. After 1000 continuous charge and discharge cycles, N_10_ still retains 94.1% of the specific capacitance value. These excellent capacitive properties are attributed to the growth of cone-shaped β-nickel hydroxide particles with high specific surface area and more complete crystallinity on the surface of the 3D nickel foam electrode.

## 5. Future Works

Nickel hydroxide is feasible as an electrode material for supercapacitors, but its conductivity needs to be further improved and its specific surface area increased. Adding conductive substances or conductive layers to the crystalized nickel hydroxide, or doping other elements to form binary or multiple transition-metal composite hydroxides, can effectively improve the capacitance characteristics of nickel hydroxide [49,50,51]. Growing nanostructures with different morphologies is an effective way to greatly increase the specific surface area. It is known from this study that the cooling rate is directly related to the research results, so it is very important to precisely monitor the cooling rate. In the future, the graph-oriented simulated annealing method proposed by Morteza Mollajafari et al. [52] can be used to make the heat treatment process smoother and more stable for temperature control and optimize the overall cooling process.

## Figures and Tables

**Table 1:** Comparison of different materials as supercapacitor electrode materials.

Electrode Material	Preparation Method	GCD Current Density (A/g)	Specific Capacitance Value (F/g)	Reference in This Work
MnO_2_/graphene	CVD, hydrothermal	0.2	333.4	[30]
MnCo-layered double hydroxide	Hydrothermal	1	1581.3	[32]
Nickel–cobalt hydroxide	CVD, hydrothermal	10	2023.5	[33]
$\mathrm{rGO}-{\mathrm{Fe}}_{3}O_{4}$	Hydrothermal (with rGO)	1.25	186.6	[34]
NiO	Electrospun	1	763	[35]
$\mathrm{Cu}/{\mathrm{Cu}}_{x}O$	Thermal reduction and oxidation with MOF	3	400	[36]
Graphene foam−ZnO	Solid-state (SS) synthesis	1	448	[37]
$\mathrm{ZnO}/{\mathrm{MnO}}_{x}$	Electrodeposition, chemical bath	1	556	[38]
Ni(OH)_2_/graphene	CVD, hydrothermal	3	539	This work

**Table 2:** Energy efficiency of the three electrode materials.

Sample Number	N_100_	N_25_	N_10_
Energy efficiency	73.4%	81.2%	90.4%

**Table 3:** Comparison of different preparation methods of nickel hydroxide as supercapacitor electrode material.

Preparation Method	Phase of Crystalline Nickel Hydroxide	GCD Current Density(A/g)	Specific Capacitance Value (F/g)	References
Hydrothermal	α	0.5	516	[45]
AAO-template-supported precipitation	α	5	833	[46]
Ultrasonication and alkalization	β	3	1171	[47]
Electrospun	β	1	763	[48]
Hydrothermal	β	3	539	In this work

## Data Availability

The data presented in this study are available on request from the corresponding author.

## References

[1] Liang C., Wang S., Sha S., Lv S., Wang G., Wang B., Li Q., Yu J., Xua X., Zhang L. (2023). Novel semiconductor materials for advanced supercapacitors. J. Mater. Chem. C.

[2] Conway B.E. (1999). Electrochemical Supercapacitors: Scientific Fundamentals and Technological Applications.

[3] Kotz R., Carlen M. (2000). Principles and applications of electrochemical capacitors. Electrochim. Acta.

[4] Zhao X., Sánchez B.M., Dobson P.J., Grant P.S. (2011). The role of nanomaterials in redox-based supercapacitors for next generation energy storage devices. Nanoscale.

[5] Frackowiak E. (2007). Carbon materials for supercapacitor application. Phys. Chem. Chem. Phys..

[6] Burke A. (2007). R&D considerations for the performance and application of electrochemical capacitors. Electrochim. Acta.

[7] Mendoza-Ponce P., John B., Schroeder D., Krautschneider W.H. Super-capacitors for implantable medical devices with wireless power transmission. Proceedings of the 14th Conference on Ph. D. Research in Microelectronics and Electronics (PRIME).

[8] Horn M., MacLeod J., Liu M., Webb J., Motta N. (2019). Supercapacitors: A new source of power for electric cars?. Econ. Anal. Policy.

[9] Al-Furjan M.S.H., Qi Z.H., Shan L., Farrokhian A., Shen X., Kolahchi R. (2023). Nano supercapacitors with practical application in aerospace technology: Vibration and wave propagation analysis. Aerosp. Sci. Technol..

[10] Végvári Z. (2019). Supercapacitors and Their Military Applicability. Honvédségi Szle..

[11] Shinde P.A., Olabi A.G., Chodankar N.R., Patil S.J., Hwang S.K., Abdelkareem M.A. (2023). Realizing superior redox kinetics of metal-metal carbides/carbon coordination supported heterointerface for stable solid-state hybrid supercapacitor. Chem. Eng. J..

[12] Lu J.L., Li J.E., Wan J., Han X.Y., Ji P.Y., Luo S., Gu M.X., Wei D.P., Hu C.G. (2020). A Facile Strategy of In-situ Anchoring of Co_3_O_4_ on N Doped Carbon Cloth for an Ultrahigh Electrochemical Performance. Nano Res..

[13] Chatterjee M., Sain S., Roy A., Das S., Pradhan S.W. (2021). Enhanced Electrochemical Properties of Co_3_O_4_ with Morphological Hierarchy for Energy Storage Application: A Comparative Study with Different Electrolytes. J. Phys. Chem. Solids.

[14] Liang R., Du Y., Xiao P., Cheng J., Yuan S., Chen Y., Yuan J., Chen J. (2021). Transition Metal Oxide Electrode Materials for Supercapacitors: A Review of Recent Developments. Nanomaterials.

[15] Bello A., Makgopa K., Fabiane M., Dodoo-Ahrin D., Ozoemena K.I., Manyala N. (2013). Chemical adsorption of NiO nanostructures on nickel foam-graphene for supercapacitor applications. J. Mater. Sci..

[16] Chen Z., Ren W., Gao L., Liu B., Pei S., Cheng H.M. (2011). Three-dimensional flexible and conductive interconnected graphene networks grown by chemical vapour deposition. Nat. Mater..

[17] Dong X., Wang X., Wang L., Song H., Zhang H., Huang W., Chen P. (2012). 3D Graphene Foam as a Monolithic and Macroporous Carbon Electrode for Electrochemical Sensing. ACS Appl. Mater. Interfaces.

[18] Ni W., Wang T., Héroguel F., Krammer A., Lee S., Yao L., Schuler A., Luterbacher J.S., Yan Y., Hu X. (2022). An efficient nickel hydrogen oxidation catalyst for hydroxide exchange membrane fuel cells. Nat. Mater..

[19] Pei P., Huang S., Chen D., Li Y., Wu Z., Ren P., Wang K., Jia X. (2019). A high-energy-density and long-stable-performance zinc-air fuel cell system. Appl. Energy.

[20] Lee D.J., Yu S.-H., Lee H.S., Jin A., Lee J., Lee J.E., Sung Y.-E., Hyeon T. (2017). Facile synthesis of metal hydroxide nanoplates and their application as lithium-ion battery anodes. J. Mater. Chem. A.

[21] Bhat M.Y., Hashmi S.A., Khan M., Choi D., Qurashi A. (2023). Frontiers and recent developments on supercapacitor’s materials, design, and applications: Transport and power system applications. J. Energy Storage.

[22] Shi F., Li L., Wang X.-L., Gua C.-D., Tu J.-P. (2014). Metal oxide/hydroxide-based materials for supercapacitors. RCS Adv..

[23] Naeem S., Patil A.V., Shaikh A.V., Shinde U.P., Husain D., Alam M.T., Sharma M., Tewari K., Ahmad S., Shah A.A. (2023). A Review of Cobalt-Based Metal Hydroxide Electrode for Applications in Supercapacitors. Adv. Mater. Sci. Eng..

[24] Nilimapriyadarsini S., Balasubramaniam S., Manab K., Lukas S.M., Ananthakumar R. (2021). Recent trends in template assisted 3D porous materials for electrochemical supercapacitors. J. Mater. Chem. A.

[25] Hu C.C., Chen W.C. (2004). Effects of substrates on the capacitive performance of RuOx·nH_2_O and activated carbon–RuO_x_ electrodes for supercapacitors. Electrochim. Acta.

[26] Hu C.C., Chen W.C., Chang K.H. (2004). How to Achieve Maximum Utilization of Hydrous Ruthenium Oxide for Supercapacitors. J. Electrochem. Soc..

[27] Hu C.C., Chang K.H., Lin M.C., Wu Y.T. (2006). Design and Tailoring of the Nanotubular Arrayed Architecture of Hydrous RuO_2_ for Next Generation Supercapacitors. Nano Lett..

[28] Sun H., Wang C., Qi Z., Hu W., Zhang Z. (2021). Nanostructure Nickel-Based Selenides as Cathode Materials for Hybrid Battery-Supercapacitors. Front. Chem..

[29] Liuqin L., Rong L., Siyu S., Liang Z., Yifan C., Naili G., Wei S., Xiaohong Z. (2006). Controllable synthesis of reduced graphene oxide/nickel hydroxide composites with different morphologies for high performance supercapacitors. J. Alloys Compd..

[30] Bai X.L., Gao Y.L., Gao Z.Y., Ma J.Y., Tong X.L., Sun H.B., Wang J.A. (2020). Supercapacitor performance of 3D-graphene/MnO_2_ foam synthesized via the combination of chemical vapor deposition with hydrothermal method. Appl. Phys. Lett..

[31] Qi J., Xu P., Lv Z., Liu X., Wen A. (2008). Effect of crystallinity on the electrochemical performance of nanometer Al-stabilized α-nickel hydroxide. J. Alloys Compd..

[32] Hsiao Y.-C., Liao C.-H., Hsu C.-S., Yougbaré S., Lin L.-Y., Wu Y.-F. (2023). Novel synthesis of manganese cobalt layered double hydroxide and sulfur-doped nickel cobalt layered double hydroxide composite as efficient active material of battery supercapacitor hybrids. J. Energy Storage.

[33] Zhang M., Wang Y., Guo X., Li R., Peng Z., Zhang W., Zheng Y., Xie H., Zhang Y., Zhao Y. (2021). High-Performance Nickel Cobalt Hydroxide Nanosheets/Graphene/Ni foam Composite Electrode for Supercapacitor Applications. J. Electroanal. Chem..

[34] Wang H., Xu X., Wang C., Neville A., Hua Y. (2021). Fundamental Insight into the Degradation Mechanism of an rGO-Fe_3_O_4_ Supercapacitor and Improving Its Capacity Behavior via Adding an Electrolyte Additive. Energy Fuels.

[35] Erdemutu E., Bai C., Ding L. (2020). Electrospun Ni-Ni(OH)_2_/Carbon Nanofibers as Flexible Binder-Free Supercapacitor Electrode with Enhanced Specific Capacitance. J. Electron. Mater..

[36] Guo Y., Chen C., Wang Y., Hong Y., Wu H., Wang K., Niu D., Zhang C., Zhang Q. (2022). Cu/Cu_x_O@C nanocomposites as efficient electrodes for high-performance supercapacitor devices. Dalton Trans.

[37] Kasap S., Kaya I.I., Repp S., Erdem E. (2019). Superbat: Battery-like supercapacitor utilized by graphene foam and zinc oxide (ZnO) electrodes induced by structural defects. Nanoscale Adv..

[38] Samuel E., Joshi B., Kim Y.I., Aldalbahi A., Rahaman M., Yoon S.S. (2020). ZnO/MnOx Nanoflowers for High-Performance Supercapacitor Electrodes. ACS Sustain. Chem. Eng..

[39] Yi X., Sun H., Robertson N., Kirk C. (2021). Nanoflower Ni(OH)_2_ grown in situ on Ni foam for high-performance supercapacitor electrodematerials. Sustain. Energy Fuels.

[40] Kim B.K., Chabot V., Yu A. (2013). Carbon nanomaterials supported Ni(OH)_2_/NiO hybrid flower structure for supercapacitor. Electrochim. Acta.

[41] Lakshmi K.C.S., Vedhanarayanan B. (2023). High-Performance Supercapacitors: A Comprehensive Review on Paradigm Shift of Conventional Energy Storage Devices. Batteries.

[42] Eftekhari A. (2017). Energy efficiency: A critically important but neglected factor in battery research. Sustain. Energy Fuels.

[43] Zhao S., Wu F., Yang L., Gao L., Burke A.F. (2010). A measurement method for determination of dc internal resistance of batteries and supercapacitors. Electrochem. Commun..

[44] Negroiu R., Svasta P., Pirvu C., Vasile A., Marghescu C. Electrochemical impedance spectroscopy for different types of supercapacitors. Proceedings of the 2017 40th International Spring Seminar on Electronics Technology (ISSE).

[45] Wiston B.R., Ashok M. (2018). Electrochemical performance of hydrothermally synthesized flower-like α-nickel hydroxide. Vacuum.

[46] Wang Y.-X., Hu Z.-A., Wu H.-Y. (2011). Preparation and electrochemical performance of alpha-nickel hydroxide nanowire. Mater. Chem. Phys..

[47] Kuo T.-R., Yen S.-C., Kubendhiran S., Yougbaré S., Lin L.-Y., Wu Y.-F. (2022). Tailoring morphology of pure terephthalic acid induced beta nickel hydroxide using ultrasonication and alkalization for efficient energy storage. J. Energy Storage.

[48] Xie M., Xu Z., Duan S., Tian Z., Zhang Y., Xiang K., Lin M., Guo X., Ding W. (2018). Facile growth of homogeneous Ni(OH)_2_ coating on carbon nanosheets for high-performance asymmetric supercapacitor applications. Nano Res..

[49] Attia S.Y., Bedir A.G., Barakat Y.F., Mohamed S.G. (2023). A two-dimensional nickel-doped bismuth-layered double hydroxide structure as a bifunctional efficient electrode material for symmetric supercapacitors. Sustain. Mater. Technol..

[50] Berrabah S.E., Benchettara A., Smaili F., Benchettara A., Mahieddine A. (2023). High performance hybrid supercapacitor based on electrochemical deposed of nickel hydroxide on zinc oxide supported by graphite electrode. J. Alloys Compd..

[51] Emin A., Li J., Dong Y., Fu Y., He D., Li Y. (2023). Facilely prepared nickel-manganese layered double hydroxide-supported manganese dioxide on nickel foam for aqueous asymmetric supercapacitors with high performance. J. Energy Storage.

[52] Mollajafari M. (2023). An efficient lightweight algorithm for scheduling tasks onto dynamically reconfigurable hardware using graph-oriented simulated annealing. Neural Comput. Appl..

